# Supplementary material for: HIVprotI: an integrated web based platform for prediction and design of HIV proteins inhibitors
Source: J Cheminform. 2018 Mar 9;10:12. doi: 10.1186/s13321-018-0266-y (PMC5845081; doi:10.1186/s13321-018-0266-y)
Supplement: Supplementary file 2 — Additional file 2. Source code of HIVProtI web server. [file 13321_2018_266_MOESM2_ESM.zip › HIVprotI_Source-code/hivprotI/weka-3-6-6/doc/weka/classifiers/functions/package-frame.html]

weka.classifiers.functions


weka.classifiers.functions

Classes 
  
GaussianProcesses
  
IsotonicRegression
  
LeastMedSq
  
LibLINEAR
  
LibSVM
  
LinearRegression
  
Logistic
  
MultilayerPerceptron
  
PaceRegression
  
PLSClassifier
  
RBFNetwork
  
SimpleLinearRegression
  
SimpleLogistic
  
SMO
  
SMOreg
  
SPegasos
  
VotedPerceptron
  
Winnow
